# Turning Up the Heat on a Hotspot: DNA Barcodes Reveal 80% More Species of Geometrid Moths along an Andean Elevational Gradient

**DOI:** 10.1371/journal.pone.0150327

**Published:** 2016-03-09

**Authors:** Gunnar Brehm, Paul D. N. Hebert, Robert K. Colwell, Marc-Oliver Adams, Florian Bodner, Katrin Friedemann, Lars Möckel, Konrad Fiedler

**Affiliations:** 1 Institut für Spezielle Zoologie und Evolutionsbiologie mit Phyletischem Museum, Friedrich-Schiller-Universität Jena, Jena, Germany; 2 Centre for Biodiversity Genomics, Biodiversity Institute of Ontario, University of Guelph, Guelph, Ontario, Canada; 3 Department of Ecology and Evolutionary Biology, University of Connecticut, Storrs, CT, United States of America; 4 Departmento de Ecologia, Universidade Federal de Goiás, Goiânia, GO, Brasil; 5 University of Colorado Museum of Natural History, Boulder, CO, 80309, United States of America; 6 Department of Botany and Biodiversity Research, University of Vienna, Vienna, Austria; 7 Hessisches Landesmuseum Darmstadt, Abteilung Naturgeschichte–Zoologie, Darmstadt, Germany; Smithsonian Conservation Biology Institute, UNITED STATES

## Abstract

We sampled 14,603 geometrid moths along a forested elevational gradient from 1020–3021 m in the southern Ecuadorian Andes, and then employed DNA barcoding to refine decisions on species boundaries initially made by morphology. We compared the results with those from an earlier study on the same but slightly shorter gradient that relied solely on morphological criteria to discriminate species. The present analysis revealed 1857 putative species, an 80% increase in species richness from the earlier study that detected only 1010 species. Measures of species richness and diversity that are less dependent on sample size were more than twice as high as in the earlier study, even when analysis was restricted to an identical elevational range. The estimated total number of geometrid species (new dataset) in the sampled area is 2350. Species richness at single sites was 32–43% higher, and the beta diversity component rose by 43–51%. These impacts of DNA barcoding on measures of richness reflect its capacity to reveal cryptic species that were overlooked in the first study. The overall results confirmed unique diversity patterns reported in the first investigation. Species diversity was uniformly high along the gradient, declining only slightly above 2800 m. Species turnover also showed little variation along the gradient, reinforcing the lack of evidence for discrete faunal zones. By confirming these major biodiversity patterns, the present study establishes that incomplete species delineation does not necessarily conceal trends of biodiversity along ecological gradients, but it impedes determination of the true magnitude of diversity and species turnover.

## Introduction

Changing patterns in plant and animal communities along elevational gradients have interested biogeographers and macroecologists since Humboldt’s pioneering studies in the Andes 200 years ago. It was long assumed that species richness would decline monotonically with elevation, reflecting decreases in temperature and primary productivity [1] However, species richness has actually been shown to peak at intermediate elevations in 70% of past investigations [1, 2]. Only 20% of prior studies have shown the predicted monotonic richness declines with increasing elevation or otherwise divergent patterns. The dominance of hump-shaped richness patterns was long overlooked due to confounding area effects [3] and because richness often peaks at relatively low elevations but not at the lowest sites as, for example, in dung beetles [4] and ants [5].

[6] found a different pattern in the Andes of southeastern Ecuador: Richness and diversity of geometrid moths showed no change along an elevational gradient from 1020m to 2677m. Although this study did not examine diversity in the lowlands or at the highest elevations, the lack of an association with elevation was novel; it has not been observed in moths at other sites, over similar partial gradients, or in other groups of organisms. For example, geometrid species richness and diversity showed a pronounced hump-shape along a gradient from 40m to 2730m in Costa Rica [7]. It has also become evident that the study area in Ecuador represents, to date, the most species-rich region for geometrid moths worldwide, with the latest regional count at 1445 species [8].

The analysis of very species-rich assemblages of tropical arthropods confronts several challenges. Among other problems, for example, the identification of species is often impeded by the lack of experienced taxonomists [9, 10, 11] and by cryptic species that cannot be discriminated morphologically [12, 13], particularly if specimens are damaged or worn. New methodological approaches can help to overcome these problems. In particular, the analysis of sequence diversity in the barcode region of the mitochondrial cytochrome *c* oxidase I gene (COI) has proven to be a powerful tool for clarifying species boundaries when combined with traditional morphospecies sorting (e.g. [14, 15, 16]), including for studies of Geometridae [17, 18].

The present study relies upon Barcode Index Numbers (BINs), a persistent species-level taxonomic registry based on the analysis of patterns of sequence variation in the barcode region, to delineate species [19]. The study does not focus on the comparison of the results from BIN analysis with those obtained through other delineation methods, such as studies employing different sequence thresholds or the Yule-coalescent (GMYC), because results from the application of these methods are usually very similar [20, 21].

The use of DNA barcoding usually increases species richness and diversity values beyond those recognized by morphological analysis, but the extent of this increase varies considerably. Studies on groups with well-developed taxonomy have typically revealed less than a 10% increase in species richness [17, 22, 23], but two-fold or greater increases have been reported in other groups, especially those with cryptic differences in morphology [24, 25]. Large changes in species counts might shift ecological or biogeographical patterns from those based on morphology alone, especially if the incidence of cryptic species varies among taxa or along environmental gradients.

Our study evaluates the impact of using differing species delineation methods to examine patterns of species richness and turnover in geometrid moths along an elevational gradient in Ecuador. We achieve this goal by comparing the results from two sampling programs that examined the diversity of these moth communities along the same gradient with identical sampling methods. In particular, this study sought to determine the extent of the increase in local richness and diversity resulting when morphological analysis was augmented with DNA barcoding. Furthermore, we examine how figures describing beta diversity shift with the two methods. Finally, the study considers how DNA barcode results may affect perceived patterns of species turnover along this elevational gradient, testing if diversity is stable with increasing elevation, as reported in the first study.

## Material and Methods

### Sampling

**Fig 1:**
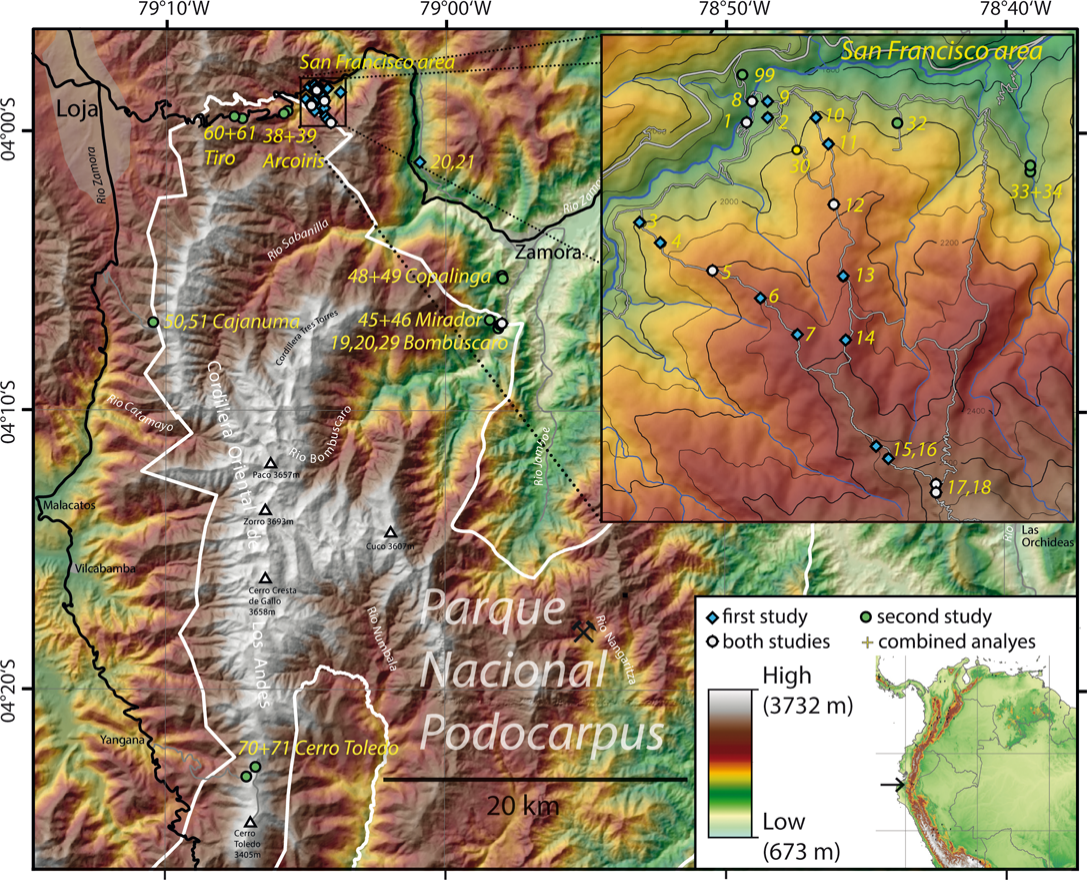
Study area with all sampling sites (first and second studies) in southern Ecuador. See S1 Table for detailed information on all sites. “+” indicates that samples from these sites were pooled for analysis.

Moths were sampled by light-trapping along a continuously forested elevational gradient in southeastern Ecuador using methods previously described e.g. by [26]. All sampling sites were in the northwestern section of Podocarpus National Park or in the adjacent Reserva Biológica San Francisco (RBSF) in the provinces of Zamora-Chinchipe and Loja (Fig 1). Collection of specimens occurred under research permits No. 023-2011-IC-FLO-DPL-MA issued by the Ministerio del Ambiente of Ecuador. None of the species is protected by international regulations such as CITES. [27] and [28] provide an introduction to the ecosystems and vegetation types in the study region. The maximum distance between the sampling sites was about 35 km, and most sites were clustered in the RBSF area in the north (Fig 1). The first study examined 22 sites [6, 29, 30] while the second sampled 28 sites, reflecting new high elevation locations that extended the elevational gradient by about 20%.

The first study, which analysed specimens collected on three field trips in 1999 and 2000, used only morphology to discriminate species. The second study examined specimens collected on three field trips in 2008, 2011 and 2013 and employed both morphology and DNA barcoding to discriminate species. Specimens from both collections were used to assess gamma diversity, and other results are analyzed both with and without the new high elevation sites.

All specimens were assigned to a subfamily, most to a genus, and some to a species. However, most species identifications were provisional because of the large numbers of undescribed species [31], and the presence of many species with close morphological similarity (see below).

Ten sites were used for quantitative analyses of local diversity, as well as eight pairs of adjacent sites from which samples were combined because local catches were small or a site was sampled just once. These merged sites were always from the same habitat type and differed less than 100 m in elevation. S1 Table details all sampling sites. All sites were undersampled, as many species expected to be present at each site were not collected, but this situation is almost inescapable in extremely species-rich assemblages [32]. Therefore, measures of richness and diversity need to be employed that reduce undersampling bias. However, we did not expect any serious qualitative effects of undersampling on the results presented in this study since all sites are similarly affected (sample coverage sensu [33], mean ± 1 SD: 0.78 ± 0.10).

Specimens in the second study were first sorted morphologically, and several representatives (when available) from each morphological group were selected for barcode analysis. A total of 3871 specimens were analysed at the Canadian Centre for DNA Barcoding, where a 658bp barcode region of the COI gene was amplified and sequenced using standard protocols [34] from DNA extracts prepared from single legs (small species) or tarsi (large species) [35]. Because of the extremely high number of species and limited resources, we used a cost-effective, stepwise approach designed to gather as many different operational taxonomic units (OTUs) as possible with a minimum number of specimens analysed. Further details, including voucher images, specimen data with GPS coordinates and collection details are publicly available in BOLD and can be accessed under dx.doi.org/10.5883/DS-ECUA. Most specimens (98%) generated a sequence >600bp and 90% delivered a 658bp read. Barcode Index Numbers (BINs) were generated from 91% of the morphospecies collected in the second study. In the remaining 9%, most species were sorted in correspondence with morphospecies recognized in the first study or from reared material. Very few species were only sorted by morphology, mostly uncommon taxa where efforts to recover a barcode from a single specimen failed. Many specimens assigned to different BINs possessed conspicuous phenotypic differences that were recognized in the initial morphological studies, but others possessed subtle differences that became apparent only after barcode analysis.

Once the barcode results were available, all specimens from the second study that were not barcoded (approximately 74% of the total catch) were sorted according to the revised species delineations. In the few cryptic species complexes where no morphological differences could be detected (< 5% of the species), specimens were assigned to a particular species based on the elevational distribution of barcoded specimens from that species complex. This approach undoubtedly led to some incorrect assignments, but such cryptic species complexes were so infrequent that they could not affect the overall results in a substantial way.

### Data analysis

We consider three sets of records: all data from the first study, all data from the second study, and all data from the second study excluding the new high elevation sites (the third approach was used only for regional comparisons). Diversity was examined at two spatial scales: individual sites (alpha), and all sites (gamma). Exponential Shannon diversity (classic formula) and Fisher's alpha were calculated for each site, and aggregated over all sites in a data set, using the software SPADE [36]). Both these parameters tend to be relatively robust even with undersampled, rich communities [37]. We additionally compared species richness across all sites with two rarefaction approaches. First, we rarefied species richness to the smallest of the three aggregated data sets (i.e., to 10,306 individuals). Second, we rarefied richness to a fixed sample coverage of 0.96. Coverage-based rarefaction makes use of information more effectively than individual-based approaches if samples are drawn from communities of very different richness [33]. At the level of local sites, species richness was estimated at a fixed coverage of 0.80. This level was selected since it meant that a similar number of sites needed to be extrapolated or rarefied. Calculations were done using iNEXT [38].

Gamma diversity was partitioned [39] into its alpha and (multiplicative) beta components (sensu [40]) based on observed species richness (diversity of the order 0) and exponential Shannon diversity (diversity of the order 1, following [41]. The relationships between local richness or diversity and elevation were examined using standard linear regression. Finally, we assessed species turnover using an unconstrained ordination method [42]. We expressed pairwise faunal similarities between sites as Chao-Soerensen index values to account for the large numbers of unseen species expected at all sites [43] (calculations performed using EstimateS 9.1.0: [44]). The resultant similarity matrix was then ordinated by means of non-metric multidimensional scaling (NMDS) using the software PRIMER [45]. Two-dimensional representations were employed due to their low stress values (< 0.09). The scores of sites along the first ordination axis were then extracted and tested for their correlations with elevation.

## Results

### Regional richness and diversity

**Fig 2:**
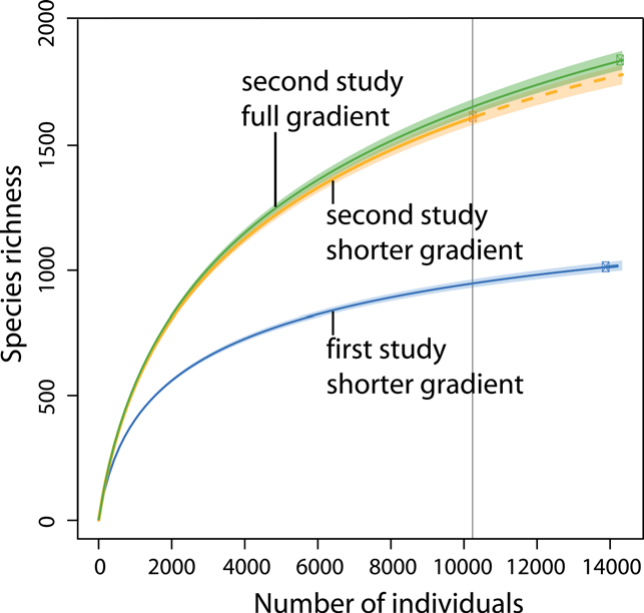
Rarefaction / extrapolation curves for regional diversity of geometrid moths in southern Ecuador. Symbols in each curve refer to observed total sample size. The grey vertical line indicates a rarefaction level of 10,306 specimens.

The number of specimens collected in the second study was just 5% higher than in the first study (Table 1). Despite this small difference, 80% more species were recognized in the second study (1857 versus 1010). The 21% increase in length of the gradient in the second study accounted for little of this difference. When the high elevation sites were excluded, and sample sizes were rarefied to 10,306 specimens for both studies, the increase in species richness was still 75% (Table 1, Fig 2). The number of singletons in the second study was very high, with 545 among 1857 species (29%). Despite the extremely high species numbers, the high proportion of singletons suggests a far higher true richness than observed values. As a consequence of the improved species resolution enabled by DNA barcoding, the single most common species (*Eupithecia duena* (Dognin)) included only 179 individuals, just 1.2% of all samples combined. By comparison, the most common species in the first study was represented by 379 individuals, but the barcode analysis revealed that the species involved, *Eois azafranata* (Dognin), is a complex. Diversity measures that are less prone to sample-size effects showed an even greater increase in the second study. Fisher’s alpha, exponential Shannon diversity as well as species richness rarefied by sample coverage were all more than twice as high in the second study (Table 1).

**Table 1:** Characteristics and results from the first and second studies of geometrid moth diversity along an elevational gradient in southern Ecuador at the regional level.

	First	Second	Change in second	Second without highest sites
Elevational range	1020–2677 m	1020–3021 m	+ 21%	1020–2677 m
Sampling expeditions	3 in 1999, 2000	3 in 2008, 2011, 2013	similar duration, but over longer period	2008, 2011, 2013
Sampling sites	22	18 (incl. 8 merged	similar, but more evenly spaced	15 (incl. 7 merged sites)
Number of specimens	13938	14603	+ 5%	10306
Observed species richness	1010	1857	+ 84%	1615
Minimum extrapolated richness (Chao 1)	1177	2350	+100%	—
Estimated richness with sample size set to 20,000 specimens	1082	2001	+ 85%	—
Overall commonest species (n)	382	179	- 53%	153
Species represented by one individual	207	545	+ 163%	527
Rarefied species number at 10,306 specimens	948.4	1,657.5	+ 75%	1,615
Rarefied species number at coverage C = 0.96	811.5	1793.8	+ 121%	1689.8
Shannon diversity	407.4	865.7	+ 113%	815.1
Fisher’s alpha	251.1	561.6	+ 124%	537.6

**Fig 3:**
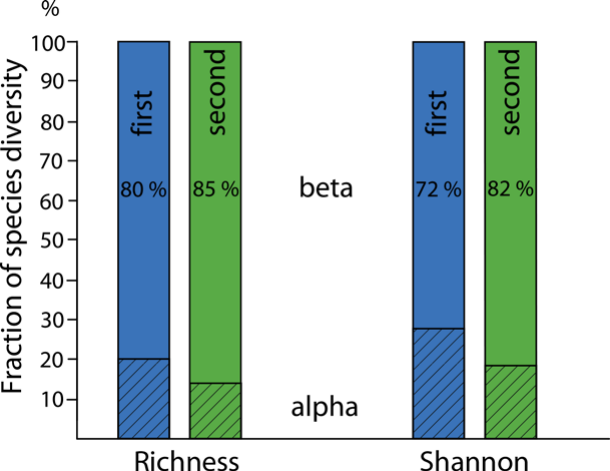
Partitioning of multiplicative gamma diversity of geometrid moths into its mean alpha and beta fractions, based on either observed species richness or exponential Shannon diversity, for the first and second studies. For both data sets, the local alpha component is, on average, smaller for species richness than for Shannon diversity. In the second data set, with far more observed species and far more species recorded as singletons, the relative contribution of the beta component is substantially larger than in the first data set, which was based on morphospecies delimitations only.

Partitioning this exceptionally high gamma diversity into its alpha and beta components revealed two patterns (Fig 3). First, when considering species richness (diversity of the order 0), the local (alpha) fraction was always considerably less than the corresponding Shannon measures (diversity of the order 1). This pattern largely reflects the impact of the many singletons, which necessarily occur at only one site. Although they strongly affect richness, they have little impact on Shannon diversity values. Second, with both measures the local (alpha) fraction substantially declined in the second study. This decline indicates that the elevational ranges of individual species were smaller in the second study, increasing the beta component of diversity relative to alpha. With species richness, the beta component increased by 43% in the new data, whereas for Shannon diversities the increase was 51%.

### Local richness and diversity

**Fig 4:**
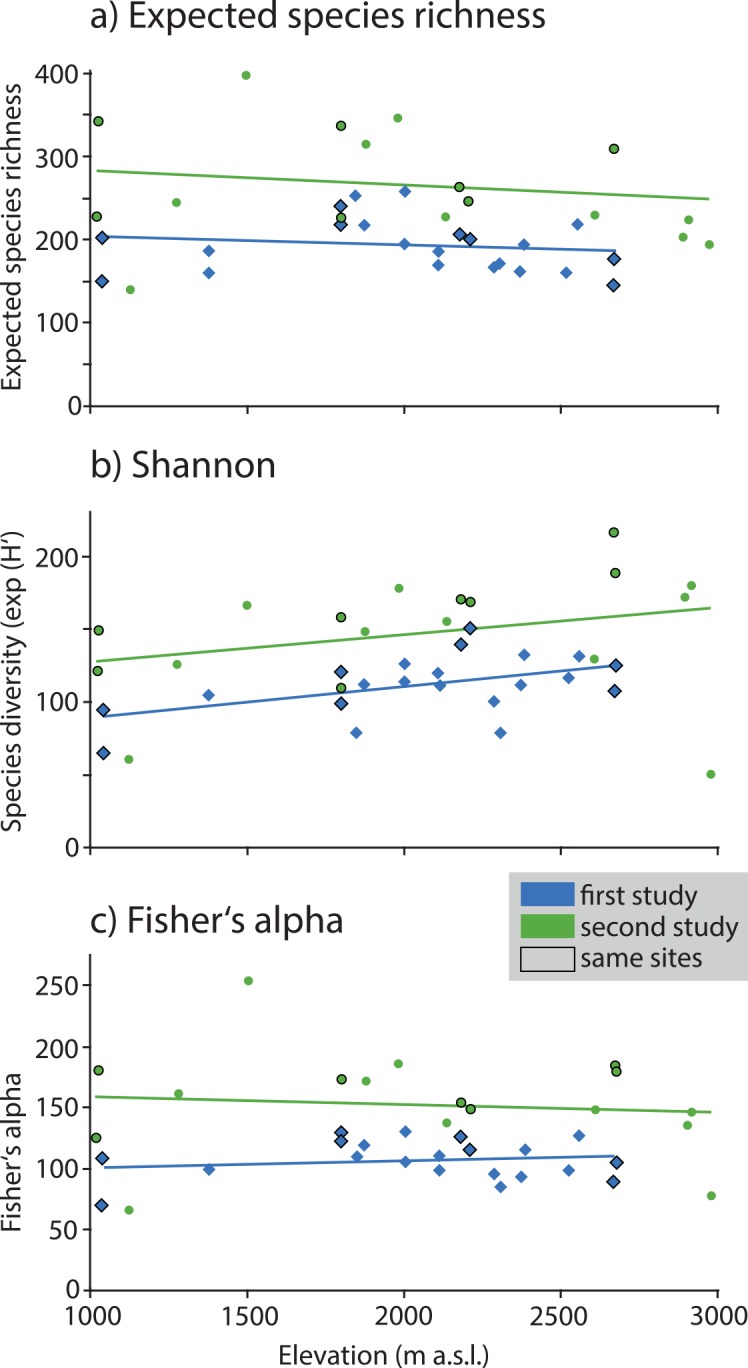
Richness and diversity patterns of geometrid moths along an elevational gradient (1000–3000 m a.s.l.) in southern Ecuador with data from the first study (blue diamonds) and the second study (green circles). The slopes of all regression lines (fitted by ordinary least squares regression) are not significantly different from zero, indicating that geometrid moth diversity is not related to elevation. Sites that were sampled in both studies are outlined in black. a) expected species richness (at coverage 0.8), b) exponential Shannon diversity, exp (H’), c) Fisher’s alpha.

When considered at the level of local sampling sites, all metrics (richness, Shannon diversity, Fisher’s alpha) were substantially greater in the second study than in the first (Table 2). When plotted against elevation, none of the three richness or diversity measures was significantly associated with elevation (Fig 4), in the second study (max(|r|) = 0.29; p > 0.24) nor in the first (max(|r|) = 0.36; p > 0.10). There was also no evidence for either a hump-shaped diversity pattern or a monotonic decline in richness or diversity with elevation. On the contrary, for exponential Shannon diversity, there was a slight (although insignificant) increase at higher elevations. Regression lines fitted to both datasets were parallel for all three measures.

**Table 2:** Diversity measures for two studies of geometrid moths along an elevational gradient in southern Ecuador at the level of sample sites. Given are means and standard deviations.

	First	Second	Increase in second
Species richness	204.8 ± 39.9	259.9 ± 80.41	+ 22%
Mean Shannon diversity	111.3 ± 20.5	147.2 ± 42.6	+ 32%
Fisher’s alpha	106.8 ± 15.5	152.9 ± 42.2	+ 43%

### Species turnover patterns

**Fig 5:**
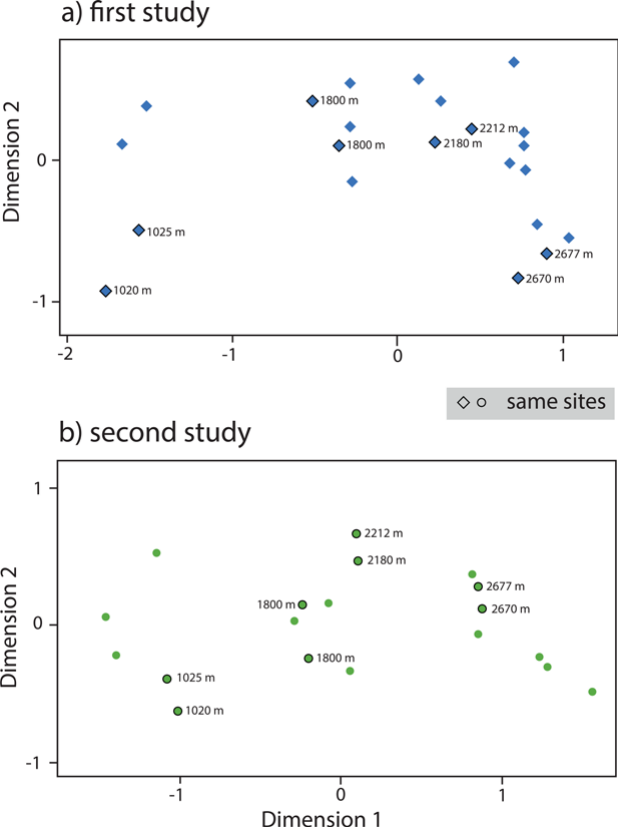
Ordination plots (by non-metric two-dimensional scaling) of species composition along an elevational gradient in southern Ecuador. Sites were largely located in the ordination according to their elevation, along dimension 1. a) first study (blue diamonds), b) second study (green circles). Sites that were sampled in both studies are outlined in black.

Unconstrained ordinations of both data sets revealed a clear elevational segregation of samples along dimension 1, which was nearly identical in strength irrespective of the approach used for species recognition (second study: r = 0.99; first study: r = 0.98, p < 0.001). A horseshoe pattern, a typical artifact of ordinations dominated by one single environmental factor (e.g., [42, 46]) was visible in both ordinations (Fig 5). Neither data set indicates the existence of discrete faunal zones, i.e. a narrow clustering of sites in reduced space.

## Discussion

### A hotspot even hotter

This study reinforces earlier evidence for the unmatched species richness of geometrid moths in southern Ecuador. [47] recognized 1266 species in southern Ecuador based on morphological studies, while [8] raised the total to 1445 putative species. The present study raises the count again to 1857 putative species, and the high fraction of singletons (almost 1/3 of the species) indicates that many more species await detection. In fact, the Chao1 estimator suggests the occurrence of 2350 ± 57 (SE) species in the elevational gradient from 1000 to 3000 m. More species are likely to occur at lower elevations and above 3000 m, and additional diurnal species from genera such as *Erateina*, *Heterusia* or *Smicropus* occur in the in the area [47]. Hence, in this small area of the Ecuadorian Andes, richness of the regional species pool of Geometridae is more than twice as high than in all of Europe (<1000 species: [48]) or the 1098 species known to occur in Borneo [49]. The application of DNA barcoding has again highlighted the extraordinary biological diversity of the tropical Andes. The new results reinforce earlier conjectures that this region is the global diversity hotspot for geometrid moths [47]. Unfortunately, it is highly threatened, by the ongoing, rapid destruction of forest habitats in the region [50].

A taxonomic revision based on past collections is in progress (GB, unpublished results) and will eventually clarify the full observed richness of geometrid moths in the study area, based on the combined results of DNA barcoding and morphology. Whether other elevational gradients in the wet tropical Andean forests of Colombia, Ecuador, Peru, and Bolivia possess comparable richness remains to be explored. Despite the increase in species richness and diversity, the observed pattern of near-constancy in species richness between 1000 m and 3000 m remains unaltered. Conclusions on the role of environmental parameters [30], on species turnover [6], and on decreasing phylogenetic diversity along the elevational gradient [8] thus remain valid. This is remarkable, since an almost doubled recognized species pool as well as a far larger fraction of species represented only as singletons would have had the potential to substantially alter emerging patterns. We conclude that cryptic diversity occurred evenly across the elevational gradient and we are not aware of comparable studies that have shown the same. From the viewpoint of ecologists and biogeographers, our observation is reassuring since the validity of earlier results is supported, even though the magnitude of richness and diversity was severely underestimated.

### Reasons for increased richness and diversity

The sharp increase in richness and diversity values in the current study is largely due to the adoption of DNA barcoding. This increase was observed both at the regional and, to a lesser extent, at the local scale. The smaller increase in local, than in regional diversity is linked to the increased and very high contribution of the beta component to regional species diversity, i.e. an even higher species turnover between sites than previously documented (Fig 3). Prior studies have established that cryptic species generate most beta diversity in European butterfly assemblages [51]. It must be emphasized, however, that undersampling (as indicated by the large proportion of singletons) tends to bias species turnover upwards, overestimating faunal differences between sites [43].

DNA barcoding has revealed many new operational taxonomic units (OTUs) in previously unrecognised species complexes of cryptic or morphologically very similar species [52, 53]. In many cases, this reflects the split of what was previously thought to be a more broadly distributed species into two or more OTUs, often with differing elevational distributions, but frequently with some overlap. Often, only one species in these species complexes has been formally described, with no morphological differential diagnosis to separate it from the rest of the OTUs in the species complex. In such cases, the assignment of this name to a particular OTU is a challenge, but advances in sequencing old type specimens can hopefully help resolve this uncertainty [24, 54].

Complexes of morphologically similar species were often lumped in the first study, because the number of available specimens for comparison was low and there was less experience in species delineation at the beginning of the project. Moreover, larger series of specimens can better be judged than single specimens. There also has been a tendency to treat species with limited morphological differences as synonyms in major reference collections such as the Natural History Museum (London). Many complexes of lumped geometrid species can nowadays be segregated in the light of combined molecular and morphological results (e.g. [55]). Often, the members of such species complexes also differ in some other dimensions of their ecological niches such as larval hostplants (e.g. [56]).

Sampling design may have contributed slightly to the observed increase in species richness. In the second study, sites were more equally spaced, and the elevational range between 1020 and 1800 m was better covered than in the earlier study. Moreover, a greater variety of habitats was sampled than before, but only in a narrow band between 1800 m and 2000 m. Preliminary observations suggest some species turnover to exist between, for example, valley and ridge sites (for tropical butterflies see e.g. [57]). The role of such small scale gradients for moths still awaits quantification. However, the extension of the upper limit of the elevational gradient by 20% played only a minor role, as demonstrated by similar values of gamma diversity with and without these sites.

### DNA barcoding as a powerful tool in biodiversity research

DNA barcoding has been validated as a powerful tool for species delineation in numerous studies, but it is particularly useful in species-rich tropical arthropod communities with their high numbers of undescribed and cryptic species. Decisions on species boundaries based on BINs or fixed sequence thresholds do not perfectly align with species boundaries when species hybridize [58] or when they are young [59]. However, such cases are clearly uncommon, as evidenced by the results of work on taxonomically well-known faunas [17, 60, 61, 62].

To our knowledge, this dataset represents the largest number of (putative) species from a single animal order to be analysed from such a small geographic region. For example, [21] studied 6500 specimens of *Trigonopterus* weevils across New Guinea and found 270 morphospecies and 324 genetic clusters an increase of 20% species with barcoding. [63] barcoded 2597 parasitoid microgastrine wasps from northwestern Costa Rica, resulting in 171 species sorted by morphological analysis and a final delineation of 312 species (an increase of 83% species with barcoding).

DNA barcoding of poorly known tropical arthropod faunas undoubtedly increases the quality and reliability of biodiversity assessments. Additional costs are involved in barcoding, but they are justified by the greater accuracy in estimation of richness and diversity values compared with studies based solely on morphology. As DNA barcode workflows usually require the deposition of voucher specimens in natural history collections, together with metadata and photographs, this wealth of information will greatly facilitate subsequent detailed taxonomic study, allowing comparisons among studies and helping to resolve the fundamental question of how many species there are on Earth.

## Supporting Information

S1 Table Supporting information on sites, species, and diversity measures. Sheet 1: All sites (first and second studies) with coordinates and elevation, sheet 2: first study: species-site matrix, sheet 3: second study: species-site matrix, sheet 4: diversity measures.
(XLSX)
